# Understanding Causation in Healthcare: An Introduction to Critical Realism

**DOI:** 10.1177/10497323221105737

**Published:** 2022-06-01

**Authors:** Erica Koopmans, Dr. Catharine Schiller

**Affiliations:** 1 School of Health Sciences, 6727 University of Northern British Columbia, Prince George, BC, Canada; 2 School of Nursing, 6727 University of Northern British Columbia, Prince George, BC, Canada

**Keywords:** critical realism, health research, philosophy, epistemology, ontology

## Abstract

Both healthcare providers and researchers in the health sciences are well rehearsed in asking the question ‘What could be causing this’? and examining beyond the surface of observable symptoms or obvious factors to understand what is really occurring with patients and health services. Critical realism is a philosophical framework that can help in this inquiry as we attempt to make sense of the observable world. The aim of this article is to introduce critical realism and explore how it can help both healthcare providers and health science researchers to better understand causation through the mechanisms that generate events, despite those mechanisms often being unseen. The article reviews foundational concepts and examples framed in the healthcare setting to make the key principles, strengths and limitations of critical realism accessible for those who are just beginning their journey with this approach.

Human health and illness are complex areas of study, and our understanding of them is typically constructed from our direct observations and experiences of events (Alderson, 2021). From what we observe, we try to make sense of, and interpret what we see happening; however, the philosophical stance we take as healthcare providers and researchers will influence our ways of thinking about these findings, and the conclusions we draw in understanding our area of study. Critical realism is a philosophical framework that is well suited to the health sciences to help us make sense of the ‘observable’ world and the ‘real’ world (Alderson, 2021). Critical realism suggests that while we may observe and experience events, they are being generated by independent, often unobservable, but still very real, mechanisms (O’Mahoney & Vincent, 2014). As healthcare providers and researchers, we are well rehearsed in looking beyond the surface of observable symptoms or factors to try and understand what is really occurring with the patients with whom we work, or the conditions and interventions which we study. The aim of this article is to introduce readers to the key tenets of critical realism, explore how it can offer healthcare providers and researchers deeper levels of explanation and understanding of causation, and examine some potential limitations of this approach.

## The Case for Critical Realism

Critical realism is not a methodology or even a theory but a way of thinking (philosophical stance), which can inform investigations into our reality (Archer et al., 2016; Oltmann & Boughey, 2012). In healthcare, critical realism can help us understand health and illness as processes that are affected by interactions between individuals and their contexts, including the agents and structures present, and help us explain what we see but also what we do not see (Alderson, 2021). In recent years, the use of critical realism by health researchers has increased as they recognize the value it provides for effectively framing, identifying and understanding complex phenomena in the healthcare sector (Schiller, 2016; Sturgiss & Clark, 2020). This approach has appeal for healthcare providers and researchers because of its recognition of the complexity of many health interventions, and its focus on explaining what works under specific conditions or contexts (Williams et al., 2016). For example, a healthcare provider may question ‘why, after trying multiple interventions that I anticipated would change the disease trajectory for my patient, am I not seeing those desired changes?’ Using critical realism, we can effectively inquire into and understand more about the unseen mechanisms that have causal influence in the situation and their effect on the patient’s health and illness (Alderson, 2021). Understanding generative mechanisms has the potential to be very meaningful when we design and evaluate new programs and services that are then transposed to another context, as it enables us to understand how and why desired change might be generated instead of just believing that it will or should happen (e.g., the effectiveness of programs or interventions).

Critical realism is also appealing given its application to various research designs and methods for data collection and analysis. This approach has been applied across broad areas of health research including in several mental health focused studies (Bergin et al., 2008; Lauzier-Jobin & Houle, 2021; Littlejohn, 2003; Martin, 2019; Sims-Schouten & Riley, 2018); rural health (Reid, 2019); as a framework for understanding smoking and tobacco control in South Africa (Oladele et al., 2013); for designing an integrated care initiative for vulnerable families in Australia (Eastwood et al., 2019); and for explaining the relationship between human rights and social determinants of health (Haigh et al., 2019).

## Foundational Concepts of Critical Realism

Critical realism emerged as a philosophical approach in the 1970s and 1980s, led by the work of Roy Bhaskar (Bhaskar, 1998, 2008) and built further by scholars such as Margaret Archer, Dave Elder-Vass, Philip Gorski, Tony Lawson and Andrew Sayer. It was introduced as an alternative philosophical framework to the positivist and interpretivist approaches being used in the natural and social sciences (Fletcher, 2017; Williams, 2003). To appreciate the value of critical realism it is important to understand how it compares to other key philosophical positions used in research and consider what it can offer that these other ways of thinking do not.

### A Focus on Ontology

Critical realism’s focus on ontology or more simply, what is real and independent of thought, awareness or knowledge of existence by humans, distinguishes it from other metatheoretical positions (Alderson, 2021). Bhaskar critiqued positivist and interpretivist philosophical frameworks because of their tendency to conflate what the world ‘is’ (*ontology*) with our experiences of it (*epistemology*) (Oltmann & Boughey, 2012; Reid, 2019). This is referred to as the *epistemic fallacy.* Positivist research is what you might think of as your ‘typical’ science experiment that uses research methods to test, observe, capture, compare and evaluate data (Hartwig, 2015). Positivism aims to identify universal laws in an objective way (Fryer, 2020). Those who use this approach consider that there is an independent, factual reality that can be discovered (Alderson, 2021). Unlike positivism, which involves searching for laws that can be generalized, interpretivist and constructivist approaches see knowledge production as fallible and theory-dependent and they tend to focus more on discourse, meaning and experiences of people (Fryer, 2020). The focus is on interpreting or constructing people’s experiences rather than discovering the actual reality which they claim is subjective to the individual (Alderson, 2021). Bhaskar argued positivist and interpretivist frameworks either limit ‘reality’ to what can be empirically studied and identified as universal laws (positivism), or view reality as entirely constructed through human discourse or experiences (interpretivism and constructivism) (Fletcher, 2017). Bhaskar criticized that research being pursued from these philosophical stances was based only on what could be observed or experienced (Clark et al., 2008). While observations and experiences might make us more confident about what exists, or what might be ‘real’, critical realists note that existence itself is not dependent on such observations (Haigh et al., 2019). For example, people have the right to health even when they are not aware they hold that right or may not have experienced it (Haigh et al., 2019). Much of the justification for using critical realism rests on the integrity of the epistemic fallacy. Critical realists need to accept this as a limitation of the framework since, when distinguishing between ontological and epistemic claims, they cannot move outside their own experiences to ‘prove’ that those distinguishing features actually exist. Positivist and interpretivist approaches do not attract the same corresponding critique as they argue that all knowledge is either objectively observed through deductive reasoning, where they look for general patterns and rules (positivism), or subjectively experienced and inductively analysed (interpretivism).

### Intransitive and Transitive Dimensions of Knowledge

Critical realism assumes the existence of an objective world, where mechanisms and structures function as *intransitive* objects, meaning they exist and act independently with powers and properties that are independent of humans but are still able to be investigated (Hartwig, 2015; Schiller, 2016). In contrast, knowledge is considered socially produced and *transitive*, meaning it is subjective; because knowledge is subjective, our understanding of phenomena can and will constantly change (Haigh et al., 2019; Vincent & O’Mahoney, 2018). Critical realists argue that we cannot just observe the world and produce knowledge about universal laws as positivists claim, without acknowledging that our beliefs, values and understanding are socially produced and changeable, meaning that knowledge is intrinsically fallible and relative. Critical realists are trying to approximate the truth of reality or the world, while remaining cognizant that all knowledge developed is fallible (Schiller, 2016). Critical realism combines observation and interpretation in a search for causation and allows for an understanding of the structural forces or mechanisms that influence our lives and generate outcomes. However, it is noted that the validity of explanation in critical realism rests upon these ontological presuppositions and we once again must assume that those presuppositions are both valid and correct.

### Stratified Reality

Critical realism suggests that reality is stratified and consists of three domains: empirical, actual and real (Fletcher, 2017). These strata can be more simply considered as experiences, events and causal mechanisms. The *empirical* layer captures our experiences, senses, feelings and observations. The *actual* refers to the events or phenomena that happen but may or may not be observed by humans. Sayer discusses that, while observability can provide confidence about what we think exists, existence itself is not dependent upon it (Sayer, 2000). The final layer is the *real*. Critical realism claims that real, but typically unseen, forces precede and generate events; these are referred to as *causal mechanisms* or *generative mechanisms* (Alderson, 2021; Hartwig, 2015)*.* Both positivism and interpretivism acknowledge the *empirical* level of trying to understand and analyse reality. Positivism also recognizes the *actual* level by acknowledging that the world does exist independently of our thoughts about that world. However, critical realism remains unique in adding the third level of *real,* yet typically unseen causal influences or mechanisms (Alderson, 2021). To explain why events, effects or outcomes occur, critical realists describe that we need to move beyond the surface of experienced and observable factors to understand what is happening underneath, at the real level (Clark et al., 2008).

Alderson (2021) supplies a helpful example, adapted here, using the condition of Type I insulin-dependent diabetes mellitus (IDDM) to demonstrate stratified reality (Table 1). To begin, you are working as a healthcare provider and a patient presents to your office describing frequent occurrences of hyperactivity as well as feelings of being weak or faint. This is experienced by that person at the empirical level. You may ask additional questions to further understand their symptoms and, as a result of this information, decide to conduct a blood glucose test. You observe from the blood test results that they have irregular blood sugar levels. The actual event that is happening is the rise and fall of blood sugar levels, but this does not explain why this is happening or what is generating this event. There could be many reasons why this individual has irregular blood sugar levels. It is not until you examine further and consider what could be causing those irregular levels that you identify that this individual’s pancreas is not secreting insulin, the hormone which converts sugar into energy. While the patient may not be aware of what their pancreas is (or is not) doing, this does not change the fact that the pancreas is indeed present and its failure to secrete insulin is causing changes to the patient’s blood sugar levels. Alderson (2021) ends this simplified life sciences example here to show how outcomes can only be understood if we dive into the context and mechanisms that generate the events we observe. Yet, we can effectively take this inquiry significantly further by using critical realism to explore *why* the pancreas is not secreting insulin. Existing research informs us that, in such situations, something will be causing the body’s immune system (which under normal conditions fight harmful bacteria and viruses) to mistakenly destroy insulin secreting beta cells of the islets of Langerhans in the pancreas (Leslie & Elliott, 1994; Lernmark & Alshiekh, 2016; Moini, 2019). Is it genetics? Is it exposure to other viruses? Is it environmental factors? What are the hidden but necessary preconditions for IDDM? Using a critical realist lens of inquiry, we may be able to better understand what is generating this outcome of irregular blood sugar levels and under what conditions this outcome will be the result.

**Table 1.** Example of Stratified Reality Using Endocrinology and Diabetes in the Life and Social Sciences. Adapted from P. Alderson (2021).

	Definition	Life sciences example	Social sciences example
Empirical	Experiences, what can be observed, sensed and interpreted	Individuals with IDDM have episodes of hypers (hyperactive) and hypos (weak and faint)	Views and experiences of individuals with IDDM, their families and healthcare providers
Actual	Events or phenomena that happen but may or may not be observable	Blood sugar levels rise during hypers, fall during hypos	Observations of daily life, interactions and events related to diabetes; number of people affected and the number of services accessed; costs of diabetes care
Real	Causal mechanisms, which, while usually unseen, are real forces in that they precede and generate the actual and empirical	The pancreas fails to secrete insulin, the hormone which turns sugar into energy. The individual requires injections of insulin to control blood sugar levels and reduce risk of severe complications	How the daily life and experience of people with IDDM may be influenced by class, ethnicity, gender, income

We can also apply this stratified reality to a social sciences example where the views and experiences of patients with IDDM, their families and their healthcare providers are observed and understood at the empirical level by asking patients about their experiences receiving health services for their condition. We could also observe their daily lives, document the number of people affected, the services accessed and the cost of care incurred to identify events associated with IDDM. However, to deeply understand events, and the ways that IDDM may be influenced by structures such as class, ethnicity, gender or income, we need to consider the real level, where unseen causal mechanisms associated with structural entities and agency are at work.

### Causal Mechanisms

As introduced above, critical realists aim to develop and provide ever-deeper levels of explanation and understanding of causal or generative mechanisms and how they work (Bergin et al., 2008). A key question in critical realism is ‘for this to occur, what does the world (or the body system) need to be like?’ (Alderson, 2021). Questions of inquiry include the following: How is the effect being caused? What triggers them? What inhibits them? (Connelly, 2001). These questions ring true as both healthcare providers and researchers. While it is important to know about a patient’s experience and the actual phenomenon that is happening, we want to find and understand the mechanisms that are producing a given effect, event or outcome (or why those mechanisms are interacting in such a way that a given event does *not* happen). This contrasts the thinking of positivists who look for cause and effect relationships using lawful patterns of thinking and interpretivist approaches who do not view causality as linear but rather as meaning constructed from human activity (Bergin et al., 2008).

Critical realism acknowledges that the relationship between mechanisms and events, despite initial appearances, is not as simple as ‘cause and effect’ (Oltmann & Boughey, 2012) and it is not necessarily linear either (cannot be inferred from a regular sequence of events) (Oladele et al., 2013). Critical realism accepts the possibility of complex causality, meaning that generative mechanisms interact in different ways and will not always play out the same as actual events or previously observed empirical experiences (Angus & Clark, 2012). Sayer (2000) provides a useful description of a critical realist view of causality:

What causes something to happen has nothing to do with the number of times we have observed it happening. Explanation depends instead on identifying causal mechanisms and how they work, and discovering if they have been activated and under what conditions (p. 14)

Therefore, for critical realists it is neither the experience nor the event itself that is the most important to identify and understand, but rather how the mechanisms are coming together in the right number, combination, time and context required to generate an outcome (Oladele et al., 2013; Schiller, 2016). Critical realism also critiques the idea that only things that are present exist (Haigh et al., 2019). Consider, for example, access to health care; when access is not present, the lack of access to health care itself may generate unmet health needs as outcomes (Haigh et al., 2019). Critical realists argue that reality, specifically social reality, is produced and changed by these generative mechanisms that are activated or not activated at any given time (Connelly, 2000). It is possible for mechanisms to exist but not generate an effect or to generate a new, different or unexpected effect (Oltmann & Boughey, 2012). Mechanisms can therefore be enabling or constraining depending on the context (Oltmann & Boughey, 2012). As critical realists, we cannot assume that they will have a particular effect but rather that their interactions will result in a tendency for an effect to occur or not occur (Oltmann & Boughey, 2012). When we conduct research using critical realism then, we are looking to identify those relatively enduring tendencies or repetitions *(demi regs* or *demi regularities)* (Hartwig, 2015)*.*

Critics of critical realism may argue that this approach to causality does not avoid the problem of induction at the level of the empirical but instead just transfers it to the level of the real. Critical realists are looking to uncover the foundational unchanging, intransitive, generative mechanisms in which to ground claims about why an event will probably happen in future if these mechanisms are present. Some will question why causal mechanisms (the real) are a better candidate for this than observations or experiences (empirical)? In other words, why is there any more reason to think that these enduring tendencies are more reliable just because they exist ‘beneath’ the empirical where it is experienced. Critiques such as these need to be considered when choosing the critical realism approach over other philosophical frameworks.

### An Open System

While we may try to create a closed system in which we can conduct an experiment, control for confounding factors, and yield universal laws about interaction between outcomes and their causes, the ‘real world’ is inevitably an open system. Patients, healthcare providers and the healthcare systems in which they exist and interact are complex and unpredictable, entangled in social contexts, behaviours and relationships which cannot be neatly classified into separate variables (Alderson, 2021). It is challenging to work in the social realm because people cannot easily be placed in the controlled environments considered necessary to truly attribute an effect or event to a cause (Oltmann & Boughey, 2012). For example, if you read in a recent research article that a new behaviour change intervention has been successful in reducing cardiovascular disease risk in a randomized control trial, you may not see the same result when you try to implement this intervention in your practice. Interventions, polices, practice guidelines and programs are frequently transposed to another context and expected to work as effectively as they worked in the context in which they were first developed or tested (Oladele et al., 2013). Critical realism recognizes the difficulties that are inherent in designing social science research and helps us to understand deterministic patterns of activity (Schiller, 2016). It acknowledges that there is a causal network of interacting forces counteracting or reinforcing each other and that outcomes depend upon the conditions in which these mechanisms will operate (Schiller, 2016). There is demonstrable value then, in identifying causal mechanisms and searching for relatively enduring tendencies or repetitions to guide us in explaining how they work, if they have been activated, and under what conditions their interactions might produce outcomes.

### Agency and Structure

In using a critical realist framework, we also need to consider agency and structure. Bhaskar (2014) and Archer (1995) explain agency and structure as separate yet interdependent entities in that neither can be ‘reduced to, explained in terms of, or reconstructed from the other. There is an ontological hiatus between society and people, as well as a mode of connection’ (Bhaskar, 2014, p. 37). Their writings on agency and structure are the basis for current theorists/practitioners to apply and adapt within a healthcare context. In the context of healthcare, agents are providers and users of health services. This includes (but is not limited to) patients, their family members and support system, healthcare providers and staff, administrators and policy makers. In experimental conditions it is typically implied that each agent involved has free will, choice or agency; in other words, they can act independently and make free choices. However, in the real world, human agency is constrained by structures, other agents and resources (Alderson, 2015). As Fryer (2020) frankly describes it, people do not just wander around, acting freely and doing whatever they want. Alternatively, if they do behave in this way, they do not usually get away with it for long. The world has social structures within which we live and, due to this, we will not often make completely individual decisions that are entirely unaffected by external influence.

Structures are powerful, objective and enduring entities that exist in and through human social relationships (Alderson, 2021). Examples of these social structures include social class, gender and race. While these structures are not typically visible (although manifestations of them might be), nor are they tangible in and of themselves, they are no less real than the law of gravity (Reid, 2019). Agents do not individually construct structures, but they will reproduce, resist, change or work within them, either through direct interaction with these structures or simply via the agent’s movement through the world (Alderson, 2021). Structures would not continue to exist without agents continuing to reproduce and transform them (Martin, 2019). Further, agents will each have their own reasons, motives, decisions, hopes and intentions (conscious and unconscious) brought to bear on the influence they wield and the choices they make; these can then be very real causal influences with effects and outcomes generated through the actions they produce, maintain and transform (Alderson, 2021; Connelly, 2000). If we are to think as critical realists, we need to be aware of our own histories and motives and how they might be affecting our experiences and observations, as well as the way in which we are interpreting the experiences and observations of others (Oltmann & Boughey, 2012), such as patients or coworkers. We should also consider how the social histories of patients or coworkers may be affecting their own experiences and observations (Oltmann & Boughey, 2012). If we persist in the belief that everyone has free will or choice, for example the agency to rise above difficult life circumstances such as poverty, abuse or discrimination, then this places the power of agency above the power of structures. It implies that agency is a single overriding power instead of acknowledging the variety and complexity of the multiple powers that will exist in an open system (Alderson, 2015). While the power of social structures is not absolute, it is immense and though some individuals may be able to overcome these powers, others may not for a variety of reasons (Alderson, 2015). It is therefore vital, when conducting social research in the realm of health sciences, to pay attention to and acknowledge these complex agency-structure relationships and interactions as much as possible. If we only look at agency, we fail to consider the impact of structures and what constraints they may have on how and why someone acts in a particular way (Martin, 2019). Conversely, if we only explore structures, we assume individuals are only influenced by these constraints and have no agency or influence (Martin, 2019).

## Next Steps for Advancing Your Practice

This article attempted to make the key principles of critical realism accessible for those who are just beginning their journey with this approach. It is a high-level introduction to critical realist concepts and supplied some examples of how critical realism can be helpful in health research, health practice inquiry, and interpretation of findings and observations. There are many more comprehensive resources available to support continued learning on this subject. While readings on philosophy can often feel dense and complex, Fryer’s (2020) *A Short Guide to Ontology and Epistemology (Why Everyone Should Be a Critical Realist)*, makes it easy to ‘wrap one’s head around’ some difficult concepts. Fryer navigates the basics of ontology and epistemology and reviews different philosophical positions through entertaining and easy to understand examples. For a user-friendly and detailed expansion on critical realism and its application for health research, Alderson’s (2021) book *Critical Realism for Health and Illness Research: A Practical Introduction* is a particularly excellent guide*.* Those interested in clarifying concepts and connecting critical realist theory and methodology may wish to read Danermark, Ekstrom and Karlsson’s (2019) recently revised *Explaining Society: Critical Realism in the Social Sciences* which includes illustrative examples of recent research, and Edwards et al. (2014) *Studying organizations using critical realism: A practical guide.* Lastly, if you are interested to dive into more complex reading in this area, *Critical Realism: Essential Readings* contains key works of many thought leaders in the field, including Archer, Bhaskar and Collier (Archer et al., 2013).

## Conclusion

Health and illness affect every aspect of our lives and are influenced by many factors, including the context, policies, behaviours and beliefs that surround us (Alderson, 2021). Patients with the same diagnosis can differ in their presentation of symptoms and how they respond to interventions. Interventions developed and studied with demonstrated efficacy in one context may fail to result in the same outcomes in another context. This article provided an overview of foundational critical realist concepts using examples from the healthcare setting. The aim was to support healthcare providers and health science researchers to consider how critical realism can help them understand causation at a deeper level and thus support more effective change, while also noting the assumptions and critiques they may encounter when using this approach. Critical realism offers many opportunities as described, including an affinity with the way many of us in healthcare see the world fitting together (O’Mahoney & Vincent, 2014). While we may observe what we think are universal laws, and experience actual events which shape our stories and guide our thinking, critical realism helps us avoid conflating what is real with our experiences. It can assist us in understanding the open system of our social world where relationship between mechanisms and events is not as simple as ‘cause and effect’, and where context, structures, and agents can interact in diverse ways to generate or constrain effects, events or outcomes. This way of thinking can help us examine beyond the surface of observable symptoms or obvious factors to understand what is really happening with patients and health services. As we attempt to make sense of the ‘real’ world and the ‘observable’ world, critical realism is a way of approaching healthcare issues that can allow us to be more successful in this endeavour.
